# Changes in *Vibrio natriegens* Growth Under Simulated Microgravity

**DOI:** 10.3389/fmicb.2020.02040

**Published:** 2020-08-28

**Authors:** Man Yin, Bingyu Ye, Yifei Jin, Lin Liu, Yan Zhang, Ping Li, Yahao Wang, Ye Li, Yanping Han, Wenlong Shen, Zhihu Zhao

**Affiliations:** ^1^Beijing Institute of Biotechnology, Beijing, China; ^2^College of Life Science, Henan Normal University, Xinxiang, China; ^3^Wuhan Frasergen Bioinformatics Co., Ltd., Wuhan, China; ^4^State Key Laboratory of Pathogen & Biosecurity, Beijing Institute of Microbiology and Epidemiology, Beijing, China

**Keywords:** simulated microgravity, Hi-C, chromosome conformation, transcriptome, SNPs

## Abstract

The growth rate of bacteria increases under simulated microgravity (SMG) with low-shear force. The next-generation microbial chassis *Vibrio natriegens* (*V. natriegens*) is a fast-growing Gram-negative, non-pathogenic bacterium with a generation time of less than 10 min. Screening of a *V. natriegens* strain with faster growth rate was attempted by 2-week continuous long-term culturing under SMG. However, the rapid growth rate of this strain made it difficult to obtain the desired mutant strain with even more rapid growth. Thus, a mutant with slower growth rate emerged. Multi-omics integration analysis was conducted to explore why this mutant grew more slowly, which might inform us about the molecular mechanisms of rapid growth of *V. natriegens* instead. The transcriptome data revealed that whereas genes related to mechanical signal transduction and flagellin biogenesis were up-regulated, those involved in adaptive responses, anaerobic and nitrogen metabolism, chromosome segregation and cell vitality were down-regulated. Moreover, genome-wide chromosome conformation capture (Hi-C) results of the slower growth mutant and wide type indicated that SMG-induced great changes of genome 3D organization were highly correlated with differentially expressed genes (DEGs). Meanwhile, whole genome re-sequencing found a significant number of structure variations (SVs) were enriched in regions with lower interaction frequency and down-regulated genes in the slower growth mutant compared with wild type (WT), which might represent a prophage region. Additionally, there was also a decreased interaction frequency in regions associated with well-orchestrated chromosomes replication. These results suggested that SMG might regulate local gene expression by sensing stress changes through conformation changes in the genome region of genes involved in flagellin, adaptability and chromosome segregation, thus followed by alteration of some physiological characteristics and affecting the growth rate and metabolic capacity.

## Introduction

Microgravity is an important environmental factor of outer space that can induce phenotypic and even genetic changes in organisms and numerous mutants have been obtained (Shi et al., 2014; Zhu et al., 2018). There are also concerns about the long term safety and capacity of humans to live and work in microgravity, particularly given the long-duration of space missions (Williams and Turnock, 2011). The effects of microgravity on microorganisms have attracted significant attention. It has been found some bacteria gain functions under conditions of [both simulated microgravity (SMG) and aircraft] (Fang et al., 1997a,b). The gain of antibiotic resistance during bacterial growth in microgravity would pose a great threat to the health of astronauts (Peter, 2015; Tirumalai et al., 2019). Over the past decades, studies of bacteria grown under microgravity have mainly examined cell motility (Benoit and Klaus, 2007; Hetrick et al., 2018), the secondary metabolism (Gao et al., 2011), and the extreme environment tolerance such as acid resistance (Peter, 2015; Mora et al., 2016), osmotic pressure resistance (Vukanti et al., 2008), oxidation resistance (Aunins et al., 2018), and antibiotic resistance (Tirumalai et al., 2019), etc.

Several studies have shown that bacteria may grow at a higher rate under SMG and spaceflight (Huang et al., 2018). Microgravity may promote the growth of non-flagella bacteria over that of flagella bacteria (Baker and Leff, 2004). Additionally, the culture media may affect the density of bacteria with rich media can increase the growth rate significantly (Klaus, 2003; Baker and Leff, 2004). Moreover, Garschagen et al. (2019) recently showed that *V. natriegens* biomass increases remarkably after 24 h at 37°C under conditions of SMG, and the final cell population can reach 60-fold that of cells grown at normal gravity (1 g). *V. natriegens* is a non-pathogenic, fast-growing marine bacterium doubling twice as fast as *Escherichia coli*, and several studies have documented that it would be the next-generation microbial chassis for the normal molecular biology and biotechnology applications, including molecular cloning (Weinstock et al., 2016; Dalia et al., 2017; Lee et al., 2019), protein synthesis (Schleicher et al., 2018; Wiegand et al., 2018; Eichmann et al., 2019), and small molecule production (Hoffart et al., 2017; Long et al., 2017), etc. The rapid growth rate of *V. natriegens* makes it a more promising engineering strain as an alternative workhorse to *Escherichia coli* (Hoff et al., 2020). Additionally, the effects of microgravity on bacterial density in stable phase are mainly achieved by shortening the lag phase and prolonging the log phase (Vukanti et al., 2008) simultaneously. Despite most research has described changes in bacterial growth properties under microgravity, the growth rate mainly depends on the microbial species and culture methods used, and some results are controversial even when using identical bacteria (Benoit and Klaus, 2007). Given the implementation of future manned space missions, understanding and evaluating the response of microbial strains to microgravity is of vital importance.

Due to the chromosome length of all organism cells far exceeds that of cells themselves by several orders of magnitude, normally DNA needs to be folded to fit inside the small cells volumes. In eukaryotes, nuclear DNA is hierarchically packaged to form individual chromatin fibers, and genome conformation of each chromosome can be further divided into multiscale structural units, namely chromosome territories, compartments, topologically associating domains (TADs), and chromatin loops. These domains are mainly organized by architectural proteins and chromatin regulators such as CTCF, cohesin, and condensin, etc., and the 3D conformation of chromatin functions in many biological processes (Van Bortle and Corces, 2012; Bonev and Cavalli, 2016; Zheng and Xie, 2019). Since most eukaryotic DNA-folding related factors are absent in bacteria, it has taken much longer to understand prokaryotes’ chromosome organizations. The bacterial genome is also folded and forms the nucleus like, namely nucleoid structure, which is mainly organized and maintained by ‘nucleoid-associated’ DNA-binding proteins (NAPs), DNA supercoiling, and transcription process. Bacterial chromosome folding is also hierarchical, from large-scale macro-domains to smaller-scale structures to regulate DNA replication and transcription (Umbarger et al., 2011; Lioy et al., 2018; Dame et al., 2020). Using genome-wide chromosome conformation capture (Hi-C) technology, the 3D genome structures of *E. coli* (Lioy et al., 2018), *Bacillus subtilis* (Marbouty et al., 2015), *Caulobacter crescentus* (Le et al., 2013), *Vibrio cholera* (Val et al., 2016), and *Mycoplasma pneumonia* (Trussart et al., 2017) have been revealed. At the scale of tens to hundreds of kilobases, the bacterial chromosome can be partitioned into chromosome interaction domains (CIDs), which are analogous to the TADs of eukaryotes. Both CIDs and TADs exhibit a high degree of self-interaction trend and are insulated from flanking regions (Umbarger et al., 2011; Le et al., 2013; Marbouty et al., 2015; Bonev and Cavalli, 2016; Lioy et al., 2018; Dame et al., 2020).

Studies on biological effect of microgravity can utilize rotating zero-head-space culture vessel devices invented by NASA. These devices exploit centrifugal force to adjust the gravity force exerted on the strain to 0.01 g to realize the state of SMG (Wang et al., 2016). Phenotypic changes induced by microgravity mainly involve epigenetic modifications, since only very few single nucleotide polymorphisms (SNPs) and small insertions and deletions (Indels) occur (Aunins et al., 2018; Tirumalai et al., 2019). As an important content of epigenetics, genome conformation is associated with gene expression and varies with the surroundings (Kong and Zhang, 2019). In this study, high aspect-ratio rotating-wall vessels (HARVs) were used to create the SMG conditions (Tryggvason et al., 2001). By continuously culturing *V. natriegens* in HARVs for 2 weeks, a faster growth rate strain was expected to emerge and screened out. Since *V. natriegens* already grows at a fast rate, we were unable to identify an appropriate method to distinguish strains with fast and extra-fast growth rates. However, a reduced growth rate mutant was obtained. Using a combination analysis involving Hi-C, transcriptome analyses, and whole genome re-sequencing, the potential mechanisms of the growth rate changes were investigated. These data could also be used to explain some principles related to the rapid growth rate of *V. natriegens*.

## Materials and Methods

### Bacterial Strains and Culture

**FIGURE 1:**
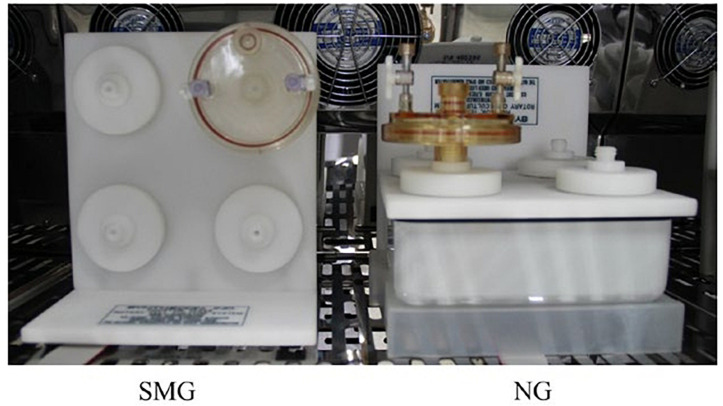
HARV bioreactors in the experimental setup and was derived from Wang et al. (2016). The bacterial cells in the HARV bioreactor were grown under the simulated microgravity (SMG) condition with its axis of rotation perpendicular to gravity or grown under the NG condition with its axis of rotation vertical to gravity when the medium was filled and the bubbles were removed.

*Vibrio natriegens* ATCC 14048 was obtained from Synthetic Genomics Inc. (La Jolla, CA, United States) and stored at −80°C in 2 × enhanced YT medium (YT) containing 25% glycerol as described in the user guide. For experiments, *V. natriegens* ATCC 14048 was grown overnight in YT medium at 37°C on a shaking incubator at 200 rpm (THZ-D, Taicang, China). SMG and normal gravity (NG) settings were established by culturing bacterial cells continuously in HARV bioreactors (Synthecon, Inc., Houston, TX, United States) as previously described (Wang et al., 2016). Figure 1, derived from Wang et al. (2016), shows the SMG cultivation achieved by rotating the bioreactor with its axis perpendicular to gravity, NG cultivation was achieved with the axis parallel to gravity (Klaus, 2003). Overnight cultures grown at 37°C with shaking at 200 rpm were inoculated at a dilution of 1:500 in the HARV bioreactors. Each bioreactor was completely filled with YT medium. Air bubbles were carefully removed. After 24 h of incubation at 37°C in HARVs with a rotation of 25 rpm, both the SMG and NG bacterial cultures were diluted into new HARVs completely filled with YT medium and incubated at 37°C and 25 rpm for another 24 h. Experimental manipulation of bacterial inoculation in the HARV bioreactors were performed for 2 weeks.

### Slower Growth Mutant Isolation and the Measurement of Growth Curves

After 2 weeks cultivation, bacterial cultures growing exponentially in YT medium in both SMG and NG were collected and stored in 1.5 mL tubes at −80°C with 25% glycerol. Then the strains from the two groups were grown overnight as described above. The cultures were inoculated at a dilution of 1:100 in 15 mL tubes with 3 mL YT medium. Cultured bacterial cells (0.5 mL) were collected, serially diluted in 1.5 mL tubes, and plated on LB agar containing 25 μg/mL kanamycin. *V. natriegens* ATCC 14048 has a natural resistance to kanamycin, and can grow slightly slowly at this concentration, greatly facilitating the identification of strains with different growth rates. Colonies that grew more slowly than SMG and wild type (WT) were selected. The above experiments were conducted both in SMG, NG, and WT for accurate comparison and selection. After screening out, the following experiments were conducted without kanamycin.

The selected colonies were grown overnight in media of YT, LB (final concentration of NaCl was 1%), and LB3 (final concentration of NaCl was 3%, the optimum salt concentration of *V. natriegens*) at 37°C with shaking at 200 rpm to reach the same OD_600_, as measured with a spectrophotometer (Bio-Rad, Hercules, CA, United States). The cultures were inoculated at a dilution of 1:100 into a new 15 mL tubes with 3 mL YT, LB3, and LB. Cell cultures (100 μL) were sampled separately every 0.5 h over the 0–10 h culture period. Bacterial growth was monitored by measuring OD_600_. The remaining collected sample cultures were suspended in phosphate-buffered saline (PBS), serially diluted, and plated on LB agar to determine the cell populations under each culturing condition. Each experiment was repeated three times.

### RNA-Seq-Based Transcriptional Analysis

Samples were collected, flash-frozen in liquid nitrogen, and treated with trizol at −80°C for RNA extraction. The RNA degradation and contamination was assessed on 1.5% agarose gels. Then RNA purity was checked using the NanoPhotometer^®^ spectrophotometer (Bio-Rad, Hercules, CA, United States). The next RNA concentration was measured using Qubit^®^ RNA Assay Kit in Qubit^®^ 3.0 Fluorometer (Life Technologies, CA, United States). RNA integrity was assessed using the RNA Nano 6000 Assay Kit of the Agilent Bioanalyzer 2100 system (Agilent Technologies, CA, United States). RNA (1 μg) was used for cDNA library construction using NEBNext^®^ Ultra^TM^ RNA Library Prep Kit for Illumina^®^ (NEB, United States). Library preparations were sequenced using an Illumina Hiseq X Ten platform and 150 bp paired-end reads were generated.

The fragments per kilobase of transcript per million fragments mapped values were calculated to determine the expression levels of transcripts. We identified differentially expressed genes (DEGs) using the following criteria: Fold-change ≥ 2 and FDR < 0.05. KOBAS (v3.0) (Xie et al., 2011) was used for pathway enrichment analysis of DEGs. KOBAS 3.0 is a server for the functional annotation of genes and proteins (Annotate module) and functional gene set enrichment (Enrichment module). The Annotate module accepts a gene list as input, including IDs or sequences, and generates annotations for each gene using pathways, and Gene Ontology databases. A two-fold change was selected as the arbitrary threshold indicating differentially regulated genes. Two replicates were generated per group.

### Whole Genome Resequencing

The slower growth mutant and WT strain genomes were sequenced as described previously (Tirumalai et al., 2017) using the TruSeq Library Construction Kit (Illumina) and compared with the reference genome. Genomic changes were identified using Burrows–Wheeler Alignment tool (BWA) with default parameters. SNPs and Indels were analyzed using GATK and VarScan, and were annotated by Annovar. The WT strains were obtained from Synthetic Genomics Inc., so their genome sequences may differ from that of the reference genome. Therefore, we also sequenced the WT strain to obtain more accurate variation information.

### Hi-C and Bioinformatics Analysis

Each sample was collected and used for a Hi-C pipeline as described previously (Lieberman-Aiden et al., 2009). Cultured strains (10^9^) were collected by centrifugation and washed using TE (Tris-EDTA buffer containing 10 mM Tris-HCl (pH 8.0) and 1 mM EDTA). Washed cells were crosslinked with 3% (final concentration) fresh formaldehyde for 10 min at room temperature (RT). Formaldehyde was quenched with 0.375 M final concentration glycine for 20 min at 4°C. Fixed cells were frozen in liquid nitrogen and stored at −80°C until use. About 1 × 10^9^ cells were suspended in 100 μL TE with 2 μL of lysozyme (Ready-Lyse Lysozyme Solution; Epicentre), and incubated at room temperature (RT) for 10 min. SDS (Sodium dodecyl sulfate) was added to the mix (final concentration 0.5%) and the cells were incubated for 10 min at RT. SDS molecules were quenched by adding 50 μL 10% Triton X-100 and the cells were incubated at RT for 10 min. Lysed cells were digested by adding 50 μL 10 × NEB buffer 2.1 (NEB), 300 μL water, and 100 U of *Sau*3AI. DNA was digested for at least 5 h at 37°C. Restriction fragment ends were labeled with biotinylated cytosine nucleotides by biotin-14-dCTP (TriLINK). Blunt-end ligation was performed at 16°C overnight in the presence of 100 Weiss units of T4 DNA ligase (Thermo, 10.0 mL final volume). After ligation, cross-linking was reversed using 200 μg/mL proteinase K (Thermo) at 65°C overnight. DNA purification was achieved using the QIAamp DNA Mini Kit (Qiagen) following the manufacturers’ instructions. Purified DNA was sheared to a length of ∼400 bp (Covaris M220). Point ligation junctions were pulled down by Dynabeads^®^ MyOne^TM^ Streptavidin C1 (Thermo Fisher) according to the manufacturers’ instructions. The Illumina sequencing Hi-C library was prepped using the NEBNext^®^ Ultra^TM^ II DNA library Prep Kit for Illumina (NEB) according to manufacturers’ instructions. Fragments between 400 and 600 bp were paired-end sequenced on the Illumina HiSeq X Ten platform (San Diego, CA, United States) and 150 bp paired-end reads were generated. Two replicates were generated for each group.

After quality filtering using Trimmomatic (version 0.38), clean Hi-C data of two biological replicates was iteratively mapped to the genome (GCA_001680025.1) using the ICE software package (version 1f8815d0cc9e). Dandling ends and other unusable data were filtered, and valid pairs were used to analyze the correlation efficiency of the two biological replicates for each sample using QuASAR-Rep analysis (3DChromatinReplicateQC v 0.0.1). Then, we pooled data from two replicates together for further analysis. A Hi-C map is a list of DNA-DNA contacts produced by a Hi-C experiment. The valid pairs after pooling were binned into 500 kb (200 kb, 100 kb, 40 kb, 20 kb, 10 kb, and 5 kb) non-overlapping genomic intervals to generate contact maps. Raw Hi-C contact maps can contain many different biases, such as mappability, GC content, and uneven distribution of restriction enzyme sites. The contact maps were normalized using an iterative normalization method to eliminate systematic biases.

We define the ‘matrix resolution’ of a Hi-C map as the locus size used to construct a particular contact matrix and the ‘map resolution’ as the smallest locus size such that 80% of loci have at least 1,000 contacts (Rao et al., 2014). The map resolution should reflect the finest scale at which one can reliably discern local features.

The contacts between 5 kb bins of intra-chromosome and inter-chromosome interactions of each sample were transferred to Ay’s Fit-Hi-C software (v1.0.1) (with parameter settings-L 20,000 –U 2,000,000 –p 2 –b 200) (Ay et al., 2014) to calculate the corresponding cumulative probability *P*-value and false discovery rate (FDR) *q*-value. After calculation, interactions in which both the *P*-value and *q*-value were less than 0.01, and contact count > 2 were identified as significant interactions.

### Combination Analysis of Contact Signals With Transcriptome

This was performed as previously described (Lioy et al., 2018). Contact maps and the transcription pattern for *V. natriegens* were generated and normalized as described above. Only RNA-seq reads with a mapping quality above 30 were conserved. Raw signal was then binned to match the binning of the corresponding contact maps and plotted along the genome. Both contact and transcription signals were smoothed with a Savitzky–Golay filter and the Pearson coefficient was determined.

## Results

### Difference in Growth Rate

**FIGURE 2:**
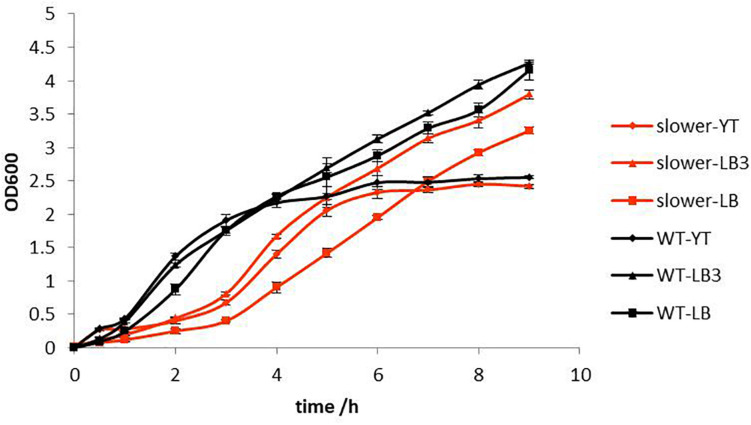
Growth rate of WT and slower growth mutant in YT, LB3, and LB medium.

After 2 weeks of continuous culturing under SMG conditions in YT media, a mutant which manifested slower growth rate was selected using 25 μg/mL kanamycin on LB plates. The growth rate of this slower growth mutant was detected under YT, LB3, and LB three different media. The results showed that the slower growth mutant would take 9 h to reach the stable phase under all conditions. As presented in Figure 2, the slower growth mutant had a longer lag phase than the WT in all three media types (Figure 2). Although most research has focused on increasing the growth rate of bacteria under different microgravity culturing conditions (Huang et al., 2018), we accidentally obtained a slower growth mutant that grew slowly in different media and salt concentrations, and can be maintained over a long culturing period.

### Changes in Transcriptome

Simulated microgravity with low-shear force changes the nutrient flows around the strains and enhances their motility in the search for more nutrients (Hetrick et al., 2018). As with prokaryotic cells, bacteria possess cell cycles which are traditionally divided into three continuous stages: a phase between cell birth and initiation of DNA replication (B period), genome duplication (C period), and a phase between completion of replication and cell division (D period) (Helmstetter et al., 1968). *V. natriegens* and *V. cholera* belong to the same genus with two chromosomes, which represents up to 10% of all bacterial species. Unlike those prokaryotes with single chromosome, these bacteria must be replicated in a well-orchestrated manner so that the two chromosomes replication terminates simultaneously and chromatin distributes evenly in the producing two daughter cells (Tirumalai et al., 2017). Therefore, genes involved in cell division events are crucial.

Bacteria utilize sensory systems to adapt to various conditions including chemoreceptors and mechanical signal transduction systems, such as chemotaxis protein, flagellin, second messenger molecules cyclic AMP (cAMP) and cyclic di-GMP (c-di-GMP) to sense and transfer the environmental mechanical signal of normal gravity and microgravity to the cell inside, and finally may through cells motility to affect gene expressions to adapt variable conditions (Hazelbauer et al., 2008; Philippe and Wu, 2010; Dufrêne and Persat, 2020).

**FIGURE 3:**
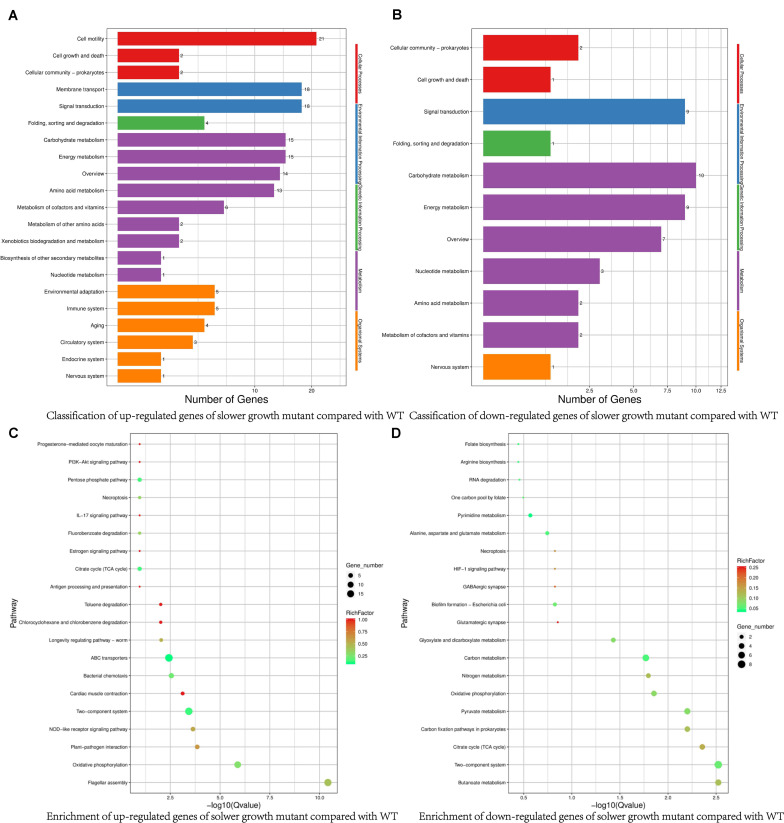
Comparison of classification and enrichment pathways of differential expressed genes in slower growth mutant. **(A)** Classification of up-regulated genes of slower growth mutant compared with WT. **(B)** Classification of down-regulated genes of slower growth mutant compared with WT. **(C)** Enrichment of up-regulated genes of slower growth mutant compared with WT. **(D)** Enrichment of down-regulated genes of slower growth mutant compared with WT.

To check the effect of SMG on *V. natriegens* gene expressions, we did the RNA-seq. Using the threshold of two-fold change, we found total of 182 DEGs, in which 123 genes and 59 genes were up- and down-regulated, respectively. Compared with WT, the up-regulated DEGs of the slower growth mutant were mainly involved in carbohydrate metabolism, signal transduction, and cell motility (Figures 3A,C and Supplementary Table S1). Signal transduction related protein such as chemotaxis protein CheV, sensor histidine kinase, methyl-accepting chemotaxis protein, MotA/TolQ/ExbB proton channel family protein and methyl-accepting chemotaxis protein were up-regulated, which were consistent with previous reports (Hazelbauer et al., 2008; Dufrêne and Persat, 2020). Besides, consistent with many reports, some flagellar assembly related genes were also significantly up-regulated. Flagellar assembly is a complex process, including cell membrane components to anchor the basal body, energy and the influx of coupling ions such as Na^+^ to rotate the flagellum, chemotaxis protein to sense gradients of attractants to change the flagellar rotation direction, etc. (Takekawa et al., 2012; Aschtgen et al., 2019). In our results, membrane protein, Na^+^/H^+^ antiporter and chemotaxis protein were up-regulated, illustrating flagella bacteria *V. natriegens* did use flagellum to sense the microgravity environment by coordinating signal transduction system to adapt the culturing conditions.

The down-regulated DEGs in the slower growth mutant included those involved in adaptive responses, the metabolism of organic acids, nitrates, amino acids, and nucleotides, and transcriptional regulation (Figures 3B,D and Supplementary Table S2). For example, there was a down-regulation of efflux pumps of the resistance nodulation division (RND) transporter related genes, which were closely associated with multidrug resistance of bacteria. *V. natriegens* is non-pathogenic bacterium, and some reports have indicated that *Vibrio* and *Pseudomonas* can utilize RND efflux pump to adapt to a new environmental conditions by changing membrane fluidity (Heipieper et al., 2003; Segura et al., 2012). There was also a down-regulation of anaerobic ribonucleoside-triphosphate reductase, formate C-acetyltransferase and nitrite reductase small subunit NirD, which were related to oxygen-limiting conditions and nitrogen metabolism. The low-shear force under microgravity causes the culturing system unable to deliver sufficient gas, and *Pseudomonas aeruginosa* has been reported to cope with apparent oxygen shortage under spaceflight conditions through denitrification (Crabbé et al., 2011). Aminobenzoyl-glutamate transporter and formyltetrahydrofolate deformylase, involved in essential vitamin folate biosynthesis (Delmar and Yu, 2016), were also down-regulated. More strikingly, chromosome segregation proteins ParB/RepB/Spo0J family partition protein was down-regulated, albeit below the 2-fold threshold (1.77-fold) (Pióro and Jakimowicz, 2020). As above described, bacteria cell cycle may be divided into three continuous stages mainly include cell birth, DNA replication and cell division. Down-regulated expression of chromosome segregation proteins may contribute to the slower growth rate. Taken together, these down-regulated genes would not favor a rapid growth rate. Under SMG or space environment, various mutations can appear, so we would further explore the genome variations and find additional supports for the slower growth mutant.

### Genome Re-sequencing and Single Nucleotide Polymorphisms Identification

Some studies have examined the nucleotide variations and its biological effect of bacteria under microgravity or under continuous culturing (Herring et al., 2006; Tirumalai et al., 2019). We compared the WT and the slower growth mutant genome sequences, and identified 117 SNPs (partially in Table 1) and 24 Indels (Table 2). Among these SNPs, 111 SNPs occurred in region located in position 1,407,507–1,414,415 of Chr. 1, which included two hypothetical proteins and one of them was down-regulated by 2.7-fold. The remaining six SNPs were involved in energy metabolism, tRNA modification (SAM-dependent methyltransferase (Kim J. et al., 2013), *N*-acetyltransferase and Signal transduction (chemotaxis protein and DUF3404 domain-containing protein). Indels were related to tRNA modification (SAM-dependent methyltransferase and tRNA modification), the same genes containing SNPs, and Phage conserved hypothetical protein (DUF2163 domain-containing protein) whose expression was down-regulated by 3.1-fold. Meanwhile, 15/24 Indels were located in position 1,407,524–1,412,121 of Chr. 1 including DUF2163 domain-containing protein, the same region where SNPs enriched. In addition, the 15 Indels were in the coding region of the down-regulated hypothetical protein. Since there presented most SNPs and Indels in Chr. 1: 1,407,524–1,412,121 and phage related protein were involved, we further scanned the genome sequence using VirSorter (Roux et al., 2015). Two regions that are likely to correspond to prophages were identified, one of which is located in this prominent region. Bacteria tend to contain multiple prophages in their chromosomes and can act as a vector for lateral gene transfer between bacteria (Canchaya et al., 2003). These sequences can encode adaptive traits to help the host enhance vitality, and most correlate with minimal doubling time (Bossi et al., 2003; Touchon et al., 2016). Prophages are involved in lysogeny, and under conditions of slow replication and poor nutrients, prophage genes can favor host survival. Under fast growth and favorable conditions, temperate phages will increase their future burst size by lysogenization, suggesting a close relationship between bacterial growth conditions and lysogeny (McDaniel and Paul, 2005). In conclusion, nucleotide variations in prophage related region might slow down the growth rate of *V. natriegens* partially. Additionally, as the above described, hierarchical structures of the genome can be considered as the basic unit of gene expression regulation, since genome variation can influence genome conformation. We would further investigate this principle in the aspect of genome organization.

**TABLE 1:** Single nucleotide polymorphism (SNPs) in slower growth mutant compared with WT.

	**Positions**	**Nucleotide type of changes**		**Peptide type of changes**	**Products**	**Differential expression**
chr1	676480	G	T	Non-synonymous SNV	Ubiquinone-binding protein	No
chr1	1939654	A	G	–	Upstream: class I SAM-dependent methyltransferase; GNAT family *N*-acetyltransferase	No
chr1	1939655	C	T	–	Upstream: class I SAM-dependent methyltransferase; GNAT family *N*-acetyltransferase	No
chr1	1990799	A	G	Non-synonymous SNV	Methyl-accepting chemotaxis protein	No
chr1	2328261	G	A	Non-synonymous SNV	Chemotaxis protein CheA	No
chr2	257319	C	T	Non-synonymous SNV	DUF3404 domain-containing protein	No

**TABLE 2:** Indels in slower growth mutant compared with WT.

	**Positions**	**Type of changes**		**Open reading frames**	**Products**	**Differential expression**
chr1	949225	T	TG	Frameshift insertion	Endopeptidase La	No
chr1	1407524	A	AAT	Frameshift insertion	DUF2163 domain-containing protein	Down (fold change = 3.1)
chr1	1407524	–	AT	Frameshift insertion	Hypothetical protein	Down (fold change = 2.7)
chr1	1407835	G	–	Frameshift deletion	Hypothetical protein	Down (fold change = 2.7)
chr1	1407841	AA	–	Frameshift deletion	Hypothetical protein	Down (fold change = 2.7)
chr1	1407844	–	GCA	Non-frameshift insertion	Hypothetical protein	Down (fold change = 2.7)
chr1	1407867	–	C	Frameshift insertion	Hypothetical protein	Down (fold change = 2.7)
chr1	1407871	–	CA	Frameshift insertion	Hypothetical protein	Down (fold change = 2.7)
chr1	1407874	TCTG	–	Frameshift deletion	Hypothetical protein	Down (fold change = 2.7)
chr1	1407878	–	AG	Frameshift insertion	Hypothetical protein	Down (fold change = 2.7)
chr1	1407881	C	–	Frameshift deletion	Hypothetical protein	Down (fold change = 2.7)
chr1	1408385	–	CT	Frameshift insertion	Hypothetical protein	Down (fold change = 2.7)
chr1	1408388	–	AC	Stopgain SNV	Hypothetical protein	Down (fold change = 2.7)
chr1	1408392	TCAG	–	Frameshift deletion	Hypothetical protein	Down (fold change = 2.7)
chr1	1412115	–	G	Frameshift insertion	Hypothetical protein	Down (fold change = 2.7)
chr1	1412117	CC	–	Frameshift deletion	Hypothetical protein	Down (fold change = 2.7)
chr1	1939661	CGCCACTT	–	–	Upstream: class I SAM-dependent methyltransferase; GNAT family *N*-acetyltransferase	No
chr1	1939672	CTAA	–	–	Upstream: class I SAM-dependent methyltransferase; GNAT family *N*-acetyltransferase	No
chr1	1939679	ATT	–	–	Upstream: class I SAM-dependent methyltransferase; GNAT family *N*-acetyltransferase	No
chr1	1939682	–	A	–	Upstream: class I SAM-dependent methyltransferase; GNAT family *N*-acetyltransferase	No
chr1	1939687	GT	–	–	Upstream: class I SAM-dependent methyltransferase; GNAT family *N*-acetyltransferase	No
chr1	1939690	AACACTTAAG	–	–	Upstream: class I SAM-dependent methyltransferase; GNAT family *N*-acetyltransferase	No
chr1	1939703	ATT	–	–	Upstream: class I SAM-dependent methyltransferase; GNAT family *N*-acetyltransferase	No
chr2	1230228	GAATAGACAACCTTTT GTCCTTTCTGATGATTAATA GATAGGCTCATATATTG TTACATTCATTCT	–	–	Downstream: ABC transporter ATP-binding protein; hypothetical protein; DUF3346 domain-containing protein	No

### Changes in Chromosome Conformation

**FIGURE 4:**
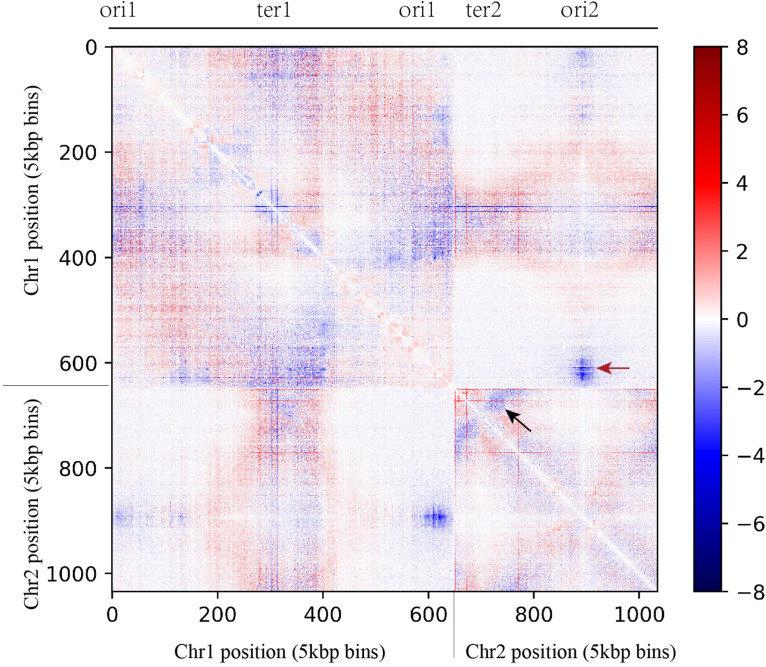
Comparison of contact map between WT and slower growth mutant obtained from asynchronous populations (YT medium, 5 kb resolution). The *x* and *y* axes represent the genomic coordinates of the reference genome (*V. natriegens* ATCC 14048, 5.2 Mb). In the heat map, the data matrix represents the log2 ratio of interaction probability between WT and slower growth mutant. The red dots show higher interaction probability in slower growth mutant and the blue dots show higher interaction probability in WT.

*Vibrio natriegens* and *V. cholera* carry two chromosomes. In *V. cholera*, initiation of second chromosome (Chr. 2) replication is triggered by the replication of a 150 bp locus positioned on the first chromosome (Chr. 1), called crtS. This allows the replication of the two chromosomes to be coordinated (Val et al., 2016). Based on the motif of crtS in *V. cholera*, we found the homologous sequence of the crtS in Chr. 1 of *V. natriegens*. This motif was located about two fifths of the length between the origin of replication (ori1) and the terminus region (ter1), consistent with the length ratio of Chr. 1 and Chr. 2. If contact between ori2 and crtS was weakened, Chr. 2 replication might initiate later, which would lead to a slower growth rate. Indeed, we did find that the interaction frequency between ori2 and ori1 decayed significantly (red arrow in Figure 4), and that there was a symmetrical structure in ter2 that also had a decreased interaction frequency (black arrow in Figure 4). Taken together, these data indicate that slow chromosome replication is at least partially responsible for the slower growth phenotype observed.

### Association of Chromosome Conformation With Transcriptome and Genome Variation

**FIGURE 5:**
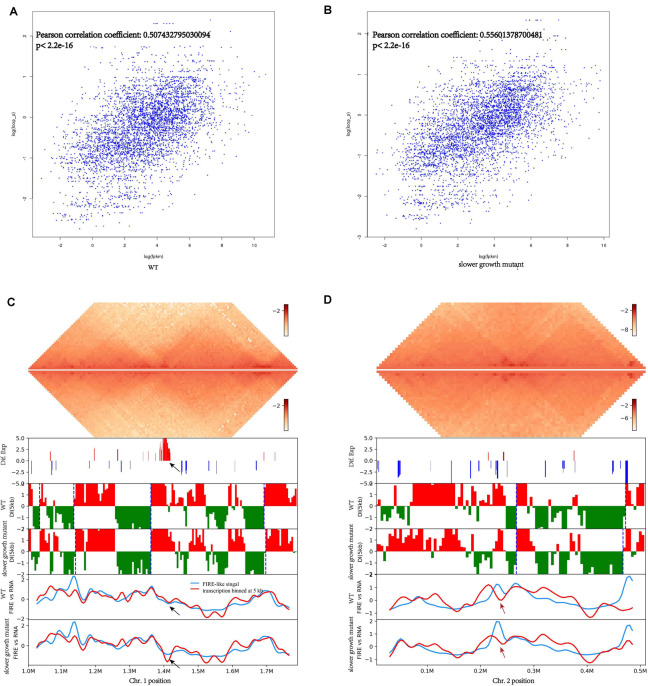
Association of genome-wide chromosome conformation and transcripts expression of *V. natriegens*. **(A)** The correlation of FIRE-like signal with transcription coverage obtained from 10 kb region analysis upstream and downstream of each locus of WT. **(B)** The correlation of FIRE-like signal with transcription coverage obtained from 10 kb region analysis upstream and downstream of each locus of slower growth mutant. **(C,D)** The contact heatmap and correlation with expressed genes of Chr. 1. The pipelines on the left from top were: WT chromosome interaction domains (CIDs), slower growth mutant CIDs, differential expression genes, DI analysis at 100 kb scale and CIDs borders are represented as blue dashed lines, transcription coverage and FIRE-like signal of WT, transcription coverage and FIRE-like signal of slower growth mutant. The black arrow showed the region of Chr. 1 with differential contact frequency. The red arrow showed the region of Chr. 2 with differential contact frequency.

Chromosome conformation and transcriptome both changed under SMG conditions. We explored whether these changes are somehow connected. We first explored the correlation of local chromatin interactions with genes expression using method searching for frequently interacting regions (FIRE) (Schmitt et al., 2016) of the genome, that is FIRE-like regions in *V. natriegens*, and found that genes in FIRE-like regions tended to have higher expression levels (Figures 5A,B). In *E. coli*, there is also a strong correlation between transcription and short-range contacts frequency (Lioy et al., 2018). Normally, short-range contacts more strongly than long-range contacts, which was a characteristic of chromosome organization (Lieberman-Aiden et al., 2009). This was observed in both the WT and the slower growth mutant, indicating that transcript expression was strongly associated with genome conformation.

Detailed further inspection of the genome-wide contact heatmap revealed a prominent region in Chr. 1 with lower interaction frequency and down-regulated genes in the slower growth mutant (Figure 5C). Genes involved in this region were mainly related to DNA integration and viral, or prophage (Table 3). *V. natriegens* contains two prophage regions. Very recently, a prophage-free *V. natriegens* variant has been established to evaluate the detrimental effects of prophages on growth and fitness of the strain, and it has showed that the prophage-free variant manifests higher tolerance toward DNA damage and hypo-osmotic stress (Pfeifer et al., 2019). Consistent with results of genome re-sequencing, this prophage related regions might be important to cell viability.

**TABLE 3:** Parts of genes in black arrow region of Chr. 1 of Figure 5C.

**Gene ID**	**Position**		**Differential expression**	**Product**
BA890_RS06375	1393096	1394310	Down	DNA integration; DNA binding; DNA recombination
BA890_RS06395	1397787	1399340	Down	Phage portal protein
BA890_RS06385	1395452	1397266	Down	Phage terminase large subunit family protein
BA890_RS06400	1399312	1401282	Down	Major capsid protein
BA890_RS06425	1402873	1403373	Down	Tail protein

Compared with the WT, one region of Chr. 2 had lower contact frequency was also found in the slower growth mutant. Genes involved were associated with anaerobic metabolism (Figure 5D and Table 4), likely because of the static surroundings of bacteria under SMG. Consequently, consistent with transcriptome data, genes expression might relate to its local genome conformation. In addition, LysR family was on both sides of this lower contact frequency region. LysR family is transcriptional regulators responsible for flexibility in diverse prokaryotic genera (Schell, 1993). In *V. cholera*, the LysR family encodes the vibriobactin receptors (Goldberg et al., 1991). Therefore, under conditions of SMG, bacteria might adjust their local genome organizations to regulate the metabolism to adapt to the environment, and hence a slower growth mutant may emerge when some essential nutrient related pathways are limited.

**TABLE 4:** Parts of genes in red arrow region of Chr. 2 of Figure 5D.

**Gene ID**	**Position**		**Differential expression**	**Product**
BA890_RS15740	191107	192813	Up	D-Lactate dehydrogenase
BA890_RS15855	214252	215001	Up	Siderophore ferric iron reductase
BA890_RS15900	223502	224332	Up	Formate dehydrogenase family accessory protein FdhD
BA890_RS15905	224373	225254	Up	LysR family transcriptional regulator
BA890_RS15910	225884	228208	Up	CbbBc protein
BA890_RS15825	209062	209385	Down	Nitrite reductase small subunit
BA890_RS15830	209573	210421	Up	Formate transporter

Combining the genome contact heatmap with the SNPs and Indels, we also concluded that genome contact numbers related to these SNPs and Indels were higher than that of the rest genome, indicating that regions with SNPs and Indels tend to be genome active regions. Taken together, slower growth mutants can arise due to genome variations that attenuate metabolic capabilities, combined with alterations in genome organization, to result in a slower growth rate.

## Discussion

There is an increasing interest in the growth rate and metabolic responses of microorganisms to the extreme environment of space. Utilizing spaceflight and ground-based SMG technologies, numerous studies have helped us understand the effects of microgravity on microbes. This study was designed to explore the mechanism of microgravity effects on bacteria growth rate. Our results show that, in the isolated slower growth mutant, most of DEGs were located in regions with chromosome structure change and were mainly related to the global transcription regulatory factors such as LysR family regulator, Flagellar assembly, signal transduction system and anaerobic metabolism. Meanwhile, a significant proportion of SNPs and Indels were found in the potential prophage regions and had higher interaction frequencies than did the average of the whole genome. Microgravity may regulate local gene expression by sensing stress changes through global regulatory factors, causing changes in genome conformation, altering some physiological characteristics, and ultimately affecting growth rate and metabolic capacity of bacteria. Since DEGs were involved in mechanical signal transduction system, these results also demonstrated that *V. natriegens* could be an ideal model to investigate the effects of microgravity on bacteria. Also, the duration of, and recovery from, the microgravity effects would be valuable information and the focus of future research. Besides, the observations of interaction frequency between ori2 and ori1 decayed significantly and chromosome segregation proteins ParB/RepB/Spo0J family partition protein down-regulated under the SMG strongly indicated further work is needed to worth of investigating the protein regulators involved in this effect, including those involved in the initiation and termination of DNA replication, such as DnaA, SeqA, MukB, and FtsZ (Reyes-Lamothe et al., 2012). Actually, in *E. coli*, nucleoid-associated protein and condensin MukBEF are involved in regulation of terminus region organization (Lioy et al., 2018).

Previous studies on *Pseudomonas aeruginosa* have concluded that when sufficient phosphate, carbon, and oxygen are available, the final cell densities are consistent with those of the motility mutant. Even in nutrient-limited conditions, cell densities are likely to increase under microgravity than they will on normal earth gravity (Kim W. et al., 2013). We tried to screen a faster growth *V. natriegens* mutant, but the original very fast growth rate made this purpose difficult. However, the slower growth mutant could provide information about the molecular mechanisms of *V. natriegens’s* fast growth rate under microgravity and normal gravity. Lots of experiments are still required to be done to investigate the underlying mechanisms for the extra fast growth rate of *V. natriegens*. A prophage-free *V. natriegens* variant shows stronger robustness (Pfeifer et al., 2019), and further microgravity researches conducted with this prophage-free variant could verify whether a more efficient strain can be obtained. As a non-pathogenic bacterium, *V. natriegens* is a promising candidate for investigating the effects of microgravity (Garschagen et al., 2019). Moreover, as it can be widely used as a host for molecular biology, *V. natriegens* can also be an alternative engineered strain to *E. coli*, which makes it much more imperative to obtain a robust strain.

## Data Availability Statement

All data needed to evaluate the conclusions in the paper are present in the paper. Additional data related to this paper may be requested from the authors. Sequence reads used for Hi-C, WGS, and RNA-seq have been submitted to NCBI with a project accession number of PRJNA624924.

## Author Contributions

MY and BY contributed to the experimental operation, data analysis, and manuscript draft writing. YJ, YL, and YH contributed to the experimental operation. LL, YZ, PL, and YW contributed to the data analysis. WS and ZZ contributed to the experimental design, supervision, and manuscript editing. All authors reviewed, revised, and approved the final report.

## Conflict of Interest

LL was employed by the company Wuhan Frasergen Bioinformatics Co., Ltd.

The remaining authors declare that the research was conducted in the absence of any commercial or financial relationships that could be construed as a potential conflict of interest.
